# PET imaging of urokinase-type plasminogen activator receptor (uPAR) in prostate cancer: current status and future perspectives

**DOI:** 10.1007/s40336-016-0197-4

**Published:** 2016-07-04

**Authors:** Dorthe Skovgaard, Morten Persson, Andreas Kjaer

**Affiliations:** 1 Department of Clinical Physiology, Nuclear Medicine & PET and Cluster for Molecular Imaging, Rigshospitalet and University of Copenhagen, National University Hospital, Blegdamsvej 9, 2100 Copenhagen, Denmark; 2 Curasight, Copenhagen, Denmark

**Keywords:** uPAR, PET, Prostate cancer, Radionuclide imaging, Theranostics, Molecular imaging

## Abstract

Overexpression of urokinase-type plasminogen activator receptors (uPAR) represents an important biomarker for aggressiveness in most common malignant diseases, including prostate cancer (PC). Accordingly, uPAR expression either assessed directly in malignant PC tissue or assessed directly in plasma (intact/cleaved forms)—provides independent additional clinical information to that contributed by PSA, Gleason score, and other relevant pathological and clinical parameters. In this respect, non-invasive molecular imaging by positron emission tomography (PET) offers a very attractive technology platform, which can provide the required quantitative information on the uPAR expression profile, without the need for invasive procedures and the risk of missing the target due to tumor heterogeneity. These observations support non-invasive PET imaging of uPAR in PC as a clinically relevant diagnostic and prognostic imaging method. In this review, we will focus on the recent development of uPAR PET and the relevance within prostate cancer imaging. Novel antibody and small-molecule radiotracers-targeting uPAR, including a series of uPAR-targeting PET ligands, based on the high affinity peptide ligand AE105, have been synthesized and tested in vitro and in vivo in preclinical murine xenograft models and, recently, in a first-ever clinical uPAR PET study in cancer patients, including patients with PC. In this phase I study, a high and specific uptake of the tracer ^64^Cu-DOTA-AE105 was found in both primary tumors and lymph node metastases. The results are encouraging and support large-scale clinical trials to determine the utility of uPAR PET in the management of patients with PC with the goal of improving outcome.

## Introduction

Prostate cancer (PC) is the most commonly diagnosed cancer amongst men in western countries [1]. The prognosis of PC is highly variable, with some PCs remaining latent disease not causing any clinical symptoms or morbidity, whereas other PCs are aggressive and associated with fast progression and high mortality [1, 2]. Due to limitations of the currently available diagnostic and prognostic tools, over-diagnosis and unnecessary treatment of indolent disease are a major issue, and novel diagnostic and/or prognostic biomarkers for PC are urgently needed [1, 3].

New sophisticated molecular imaging modalities with multiparametric magnetic resonance imaging (mpMRI) and positron emission tomography (PET) using ^18^F-fluoro-deoxy-glucose (FDG), radiolabeled choline, and alternative radioligands, such as gastrin-releasing peptide receptor (GRPR)-targeting ligand and prostate-specific membrane antigen (PSMA)-targeting ligand, are currently investigated for all aspects of PC, including diagnosis and localization, staging, active surveillance, prognosis, and monitoring recurrence [1].

In this review, we will focus on PET imaging of a new promising molecular target; urokinase-type plasminogen activator receptor (uPAR) in PC as a clinically relevant diagnostic and prognostic imaging biomarker with the possibility of distinguishing indolent tumors from the invasive phenotype. The majority of references for this review were found by searching PubMed for “uPAR”, “urokinase-type plasminogen activator receptor”, “prostate cancer”, and ‘PET’ or ‘positron emission tomography’. Additional references were also incorporated on the basis of the author’s experience in basic research within uPAR or related fields as well as by cross-referencing.

## uPAR and the aggressive phenotype

**Fig. 1:**
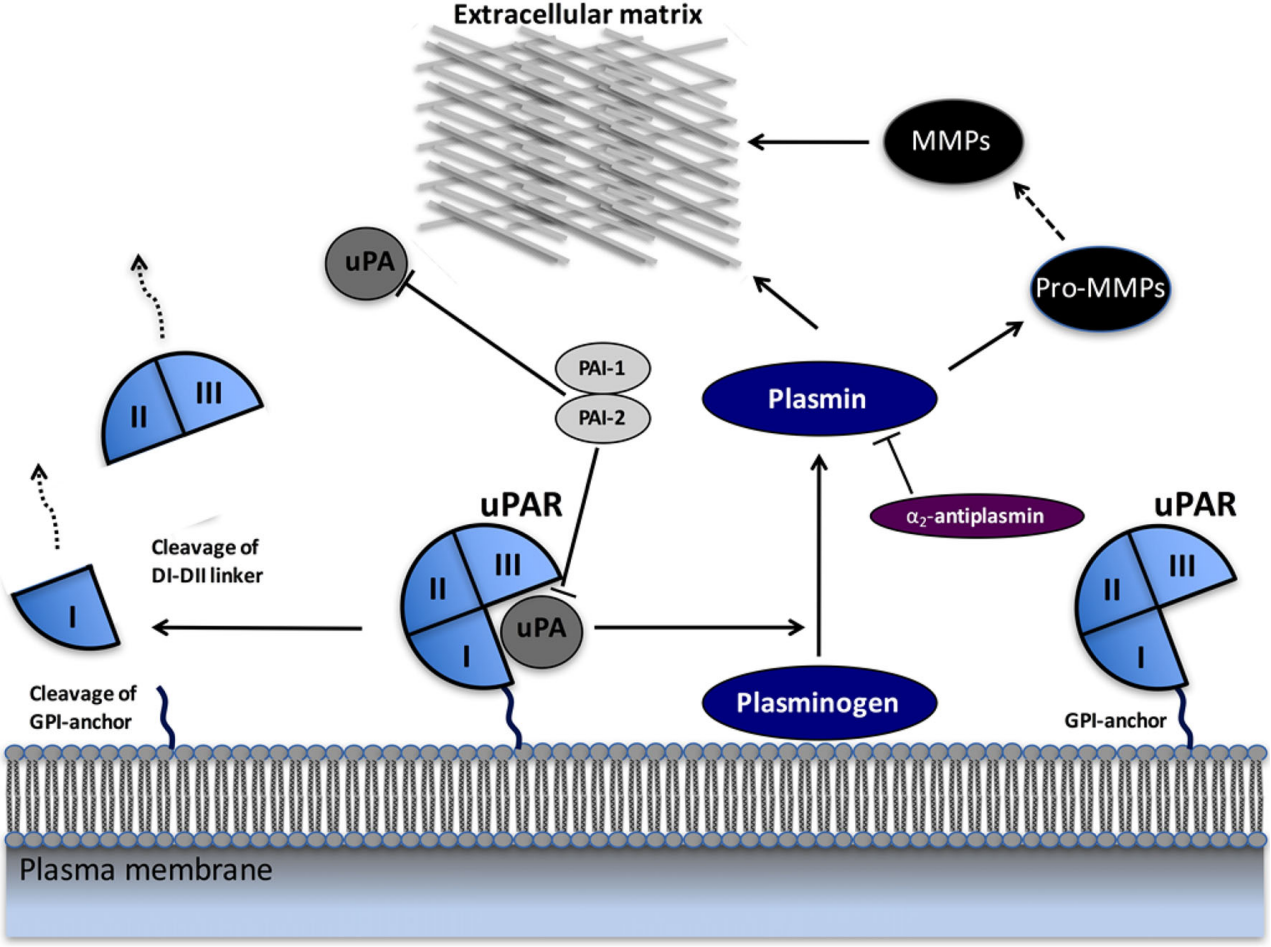
Schematic overview of the uPA/uPAR system. Urokinase-type plasminogen activator receptor (uPAR) is tethered to the cell membrane with a glycosylphosphatidylinositol (GPI) anchor and binds the protease urokinase-type plasminogen activator (uPA). uPA cleaves plasminogen, generating the active protease plasmin. Plasmin cleaves and activates matrix metalloproteases (MMPs). Both plasmin and MMPs degrade many extracellular matrix (ECM) components and thereby promote cancer invasion and metastasis. The proteolytic activities of uPA and plasmin are inhibited by PAI1, PAI2, and α2-antiplasmin. When uPA is bound to uPAR, there is cleavage between the GPI-anchor and the uPAR, releasing suPAR into the vascular system, and uPA also cleaves uPAR in the linker between its first and second domains (D1 and D2), generating a soluble D1 fragment and a membrane-associated D2–D3 fragment

The urokinase plasminogen activator (PA) system plays a key role in the pericellular proteolytic activity which is required for tissue remodeling during normal physiological conditions, such as wound healing and initiation of angiogenesis, but also in pathophysiologically processes, such as cancer invasion [2–7] (Fig. 1). The PA system consists of the serine protease urokinase-type plasminogen (uPA), its glycosylphosphatidylinositol (GPI)-anchored cell membrane receptor (uPAR), and two specific inhibitors PAI-1 and PAI-2. uPA binds with high affinity to uPAR and, consequently, converts plasminogen to active plasmin, which activates several proteases related to the degradation of extracellular matrix proteins and basal membranes, thereby facilitating cancer cell invasion and metastasis [8]. It has become increasingly clear that PA not only is central in proteolytic degradation of extracellular matrix but also affects multiple other aspects of the tumor progression and development by eliciting tumor-associated processes, such as cell proliferation, cell adhesion and migration, chemotaxis, and cell survival through interactions with co-receptors to relay intracellular downstream signaling pathways. Integrins, G-protein-coupled receptors, and growth factor receptors are found to directly interact with uPAR, and are assumed to serve as co-receptors for uPAR-mediated PAR-mediated downstream signaling and/or activation pathways [9].

Using various biochemical assays; immunohistochemistry (IHC), tissue micro-arrays, and reverse transcriptase-polymerase chain reactions (PCR), uPAR expression can be assessed directly in tumor specimens and is particularly high in cancer cells at the very front of the invasive tumor as well as in tumor-associated stromal cells, such as fibroblasts and macrophages [10–12]. High-tumor expression of uPAR has shown to predict adverse outcome in a wide variety of malignancies, including breast, colorectal, pancreatic, and PC [5].

In addition, uPAR can be cleaved from the membrane, and high levels of soluble uPAR and/or various uPAR forms in the blood have been reported in a number of cancers. Intact uPAR(I–III) can be cleaved by uPA, releasing domain I [uPAR(I)], while leaving uPAR(II–III) on the cell surface. Both of the glycolipid-anchored uPAR forms [uPAR(II–III) and uPAR(I–III)] can be shed from the cell membrane, resulting in three soluble uPAR forms [uPAR(I–III) and uPAR(II–III) as well as uPAR(I)] detectable in the blood [13]. Interestingly, the cleaved soluble uPAR forms have been demonstrated to be independent prognostic markers in various types of cancer [14], such as colorectal [15, 16], breast [17], lung [18], and PC [19]. However, measurement of plasma levels of uPAR (intact/cleaved domains) will always only be an indirect indicator for the expression level in the tumor. Moreover, the lack of correlation between tumor tissue uPAR expression and the level of secreted different forms of uPAR [20], together with the fact, the majority of cancer patients have uPAR levels within the reference interval of healthy individuals [21], further complicate the information achievable. This is, perhaps, the main reason for the lack of routine clinical use of plasma uPAR measurements. It seems that localized measurements, encompassing the heterogeneity, in the tumor and in the local microenvironment, are necessary for optimal uPAR-based diagnostic and prognostic information.

## uPAR and prostate cancer

Compared to other malignant diseases, such as breast cancer and colorectal cancer, the role of uPAR expression in PC is less well investigated and the majority of studies are based on relatively small patient populations (<200 patients) focusing on blood levels of either intact or cleaved forms of uPAR. Indeed, only few studies have attempted to measure expression of uPAR directly in prostate tumor specimens using the established biochemical techniques.

In general, uPAR IHC on PC tissue (biopsies or surgical specimens) has demonstrated increased uPAR expression in PC [22–25]. Examples of such studies include the use of standard IHC on tissue micro-arrays, where Cozzi et al. [25] found uPAR overexpression in primary PC cells, surrounding tumor-associated stromal cells and lymph node metastases, but not in normal prostate tissue. uPAR expression was highly related to disease progression and tumor differentiation, including Gleason score. An association between high uPAR detected by IHC and relevant pathological and clinical parameters, such as high Gleason score, advanced tumor stage, positive lymph node status, and incomplete tumor resection has also been confirmed in other studies [24, 26]. However, in the study by Cozzi et al. [25] and in a more recent study by Gupta et al. [26], no difference in biochemical free survival was demonstrated, possibly explained by short follow-up with only few patients experiencing biochemical progression. Importantly, others have successfully found significant impact of uPAR valuated by IHC staining of tumor specimens, on the prognosis of PC patients [24].

Similar to the immunohistochemical analysis, uPAR expression at mRNA level has only been investigated in a limited number of studies and only in small populations of PC patients, using either in situ hybridization or real-time quantitative PCR (qPCR) [22, 23, 27, 28]. Riddick et al. [28] used qPCR and found increased mRNA expression in malignant tissue samples from patients with PC compared to non-malignant samples from patients with benign prostatic hyperplasia with statistically significant positive correlations with Gleason score. However, this could not be confirmed in the study by Al-Jabani et al. [27], where increased mRNA expression levels of uPA and PAI-1 and not uPAR were found in PC tissue compared to benign prostate hyperplasia and normal prostate tissue.

As already mentioned, the majority of studies investigating implications of uPAR in PC diagnosis and prognosis have focused on the assessment of circulating soluble uPAR. In line with this, serum from patients with PC contains elevated levels of soluble uPAR compared with patients with benign prostatic hypertrophy and healthy controls [29]. In addition, pre-operative circulating total uPAR levels were found to be higher among patients with higher biopsy Gleason grade, extraprostatic extension, and lymph node positive disease after radical prostatectomy, and, indeed, PC patients with bone metastasis exhibited significantly higher uPAR levels compared with patients with localized disease or patients with lymph node metastasis [30]. Furthermore, studies have found significantly lowered overall survival rate of PC patients with high plasma levels of uPAR compared with low serum uPAR levels [27, 29]. In the most recently published study, the plasma levels of the cleaved uPAR forms, uPAR(I–III) + uPAR(II–III) and uPAR(I) levels, were significantly higher, while level of intact uPAR(I–III) did not differ, in hormone-naive and castrate-resistant patients compared with patients with localized disease, highlighting that analysis of the cleaved forms might be superior and, thus, provide additional prognostic and predictive information [31].

Although no definite conclusion can be drawn, the majority of studies, although based on relatively small populations, find uPAR expression, either assessed directly in the malignant PC tissue or in plasma (intact/cleaved forms), to be a largely independent analytical variable, conceivably offering clinical information that is different from and additive to that contributed by PSA, Gleason score, and other relevant pathological/clinical parameters. These observations highlight and support that non-invasive imaging of uPAR in PC, with the possibility of distinguishing indolent tumors from the invasive phenotype, could become a clinically relevant diagnostic and prognostic imaging biomarker, as also identified by different authors [6, 32].

## uPAR PET imaging

One of the major challenges when assessing uPAR expression directly in tumor specimen is intra-tumor heterogeneity. This is of special importance in PC, which is recognized as often being multifocal disease with a broad spectrum of clinical, pathologic, and molecular characteristics, emphasized by the routinely used 12-core biopsy protocol for diagnosis of PC [33]. In this respect, non-invasive molecular imaging by PET offers a very attractive technology platform, which can provide the required information on the global expression profile or function of the target, such as uPAR, without the need for invasive procedures [3] and the risk of missing the target due to tumor heterogeneity.

Detailed insight into the molecular basis underlying the interactions between uPAR and its ligand uPA has been obtained by X-ray crystallography and surface plasmon resonance studies. Importantly, these structural studies also defined possible target sites in uPAR for small molecules, which have led the development of a series of small peptides applicable for non-invasive molecular imaging of uPAR expression in vivo by positron emission tomography [34].

In a uPAR PET proof-of-concept study [35], one of these peptides, AE105 [36], conjugated with the metal chelator DOTA in the N-terminal and labeled with ^64^Cu was used. MicroPET imaging of mice-bearing uPAR-positive U87MG human glioblastoma and uPAR-negative MDA-MB-435 human breast cancer xenografts was used to illustrate the ability to specifically detect human uPAR. A high accumulation in the uPAR-positive U87MG xenograft tumor (10.8 ± 1.5 % ID per g) compared with the uPAR-negative MDA-MB-435 xenograft tumor (1.2 ± 0.6 % ID per g) was found 4.5 h after injection. The specificity of the tracer was further validated by comparing the uptake of a non-binding variant of the peptide in the uPAR-positive U87MG xenograft and by performing a blocking experiment using excessive pre-dose of non-labeled peptide resulting in reduced tumor uptake, thus illustrating the specificity of ^64^Cu-DOTA-AE105 for non-invasive PET imaging of uPAR [35].

**Fig. 2:**
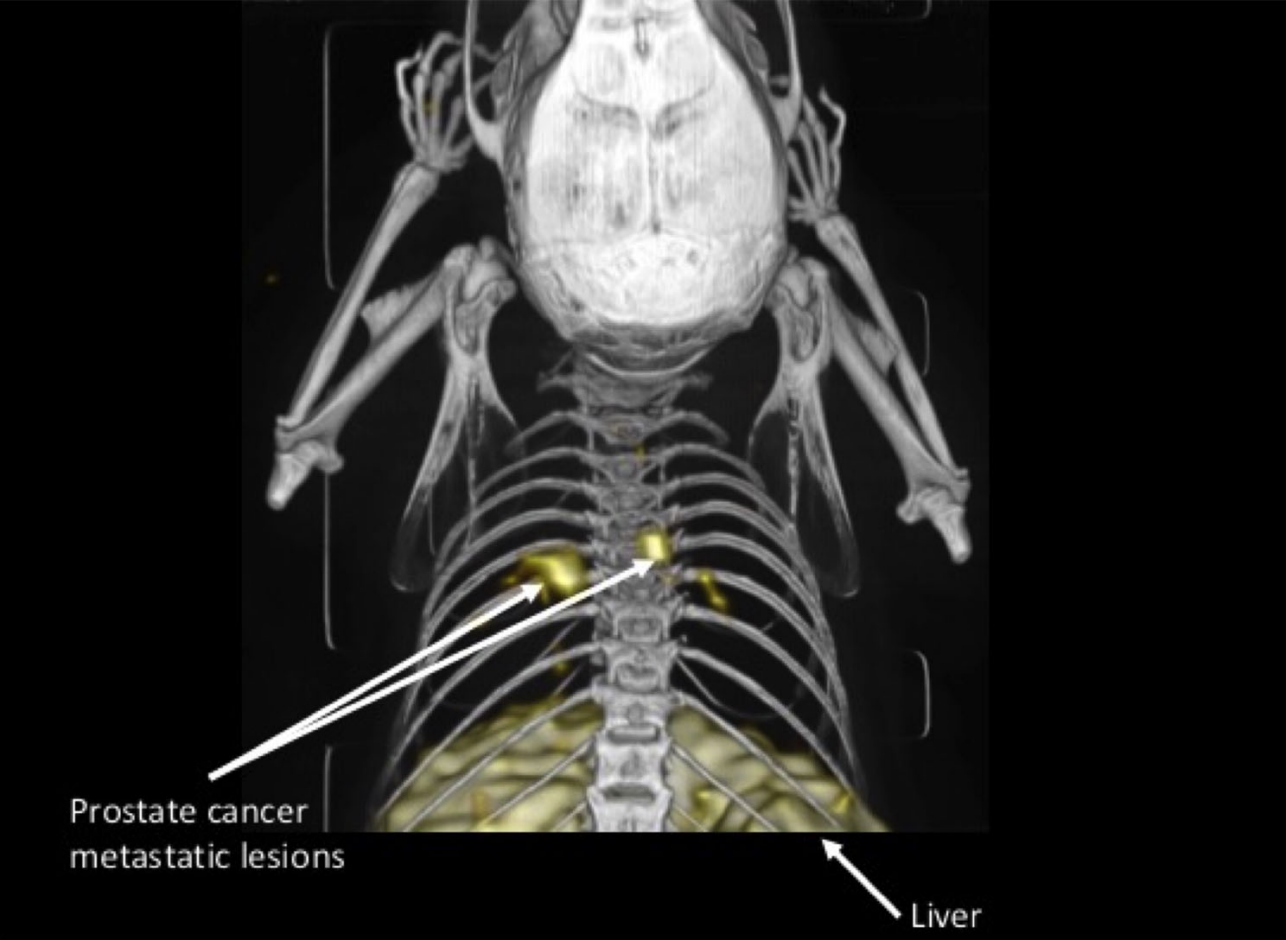
uPAR PET imaging of small metastatic lesions. In a mouse model of disseminated human prostate cancer and in this mouse model of disseminated prostate cancer, C-3 M-LUC2 cells are inoculated by intracardiac injection to mimic intravascular dissemination and subsequent systemic establishment of metastatic disease. As the PC-3 M-LUC2 cell-line is stably transfected with luciferase, the formation of small metastatic lesions can be followed with bioluminecence imaging (BLI). By comparison all tumors, lesions identified on BLI scanning were also identified on uPAR PET on day 31 post initiation. *Arrows* indicate metastatic lesions with clearly visualized uptake of ^64^Cu-DOTA-AE105 and unspecific uptake of ^64^Cu in the liver Adapted with permission from [48]. Copyright 2014 American Chemical Society

In our group, the focus has also been on AE105 in our efforts to develop a uPAR-targeting PET ligand. We have investigated the use of different metal-binding chelators and different isotopes, including ^64^Cu, ^68^Ga, and ^18^F [4, 37–40] (Fig. 2). Importantly, in our first experience, we found a significant correlation between tumor uptake of ^64^Cu-DOTA-AE105 on microPET images of human tumor xenografts and uPAR expression level in the tumor tissue [37]. However, our results also revealed a relatively high accumulation of ^64^Cu in the liver, a known site for ^64^Cu accumulation, and a well-established indirect marker of instability of ^64^Cu-based ligands in rodents [41, 42]. Therefore, two improved metal chelators (^64^Cu-CB-TE2A-AE105 and ^64^Cu-CB-TE2A-PA-AE105) based on cross-bridge cyclam N-conjugated to the AE105 were developed and tested both in vitro and in vivo in preclinical mice cancer models. In particular, ^64^Cu-CB-TE2A-PA-AE105 exhibited an improved tumor-to-liver ratio. In line with this and based on the fast tumor uptake observed in our study, we hypothesized that the use of ^68^Ga instead of ^64^Cu could maintain tumor uptake and reduce the non-specific uptake in non-target tissue, especially the liver. Furthermore, the half-life of ^68^Ga more resembles the biological half-life of our peptide-based ligand, and as ^68^Ga is a generator-based radionuclide, this could make our ligand more widely used in PET centers. The results of using ^68^Ga showed a significant reduction in liver uptake as expected for both ^68^Ga-DOTA-AE105 and ^68^Ga-NODAGA-AE105 [38]. However, this reduction was also accompanied by a reduction in tumor uptake and a lower tumor-to-kidney ratio, compared with ^64^Cu-DOTA-AE105. Later, we tested ^18^F-labeled uPAR PET ligand, ^18^F-AlF-NOTA-AE105, and effectively visualized non-invasively uPAR-positive PC in mice models with high tumor-to-background ratio. Ex vivo uPAR expression analysis on extracted tumors confirmed human uPAR expression that correlated close with tumor uptake of ^18^F-AlF-NOTA-AE105. In our latest effort to develop a clinical uPAR PET ligand, ^64^Cu-NOTA-AE105 and ^68^Ga-NOTA-AE105 were developed and investigated in a human orthotopic glioblastoma model in mice [40]. Again, uPAR expression levels correlated with uPAR radiotracer uptake in resected glioblastoma tumors.

## uPAR PET imaging in patients with PC

**Fig. 3:**
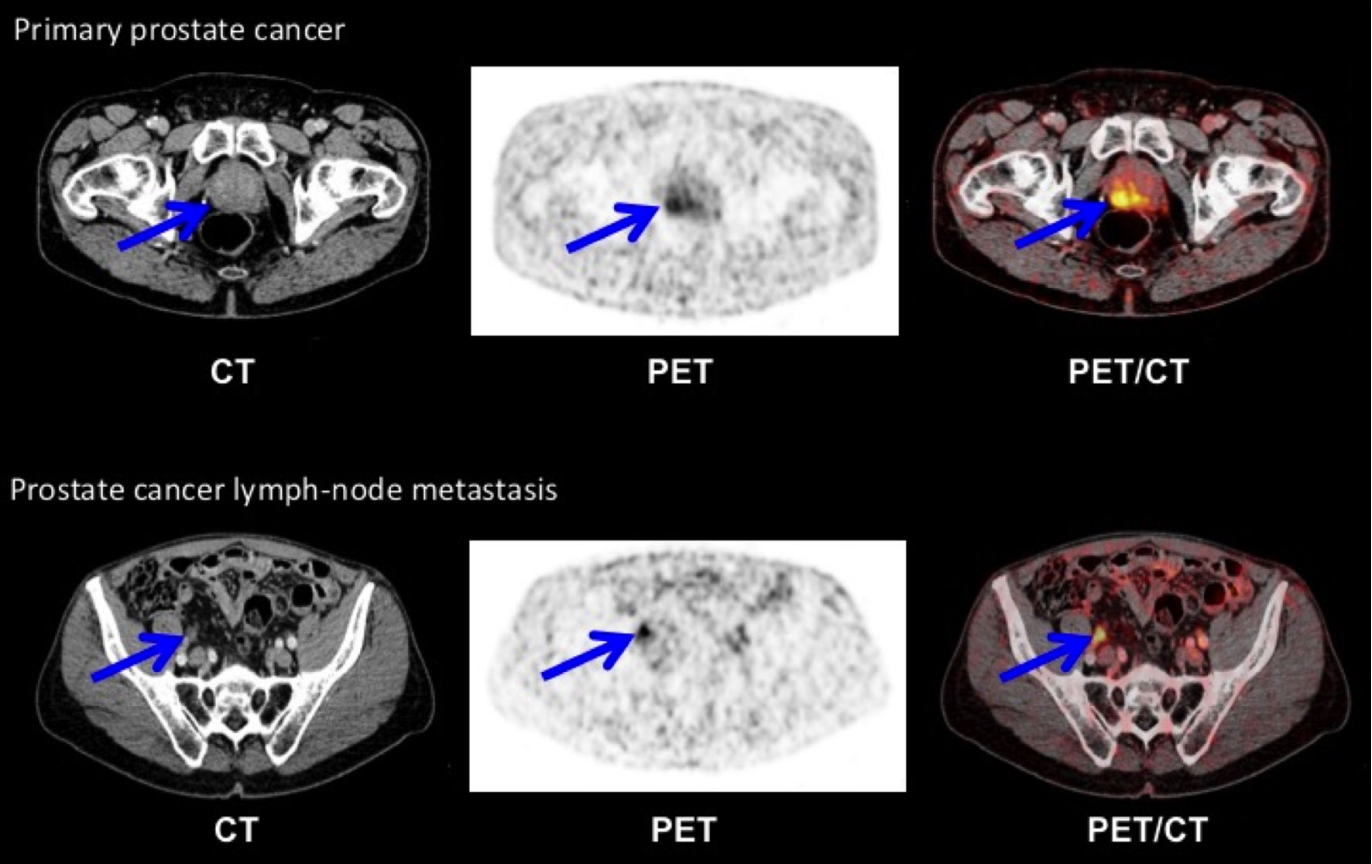
uPAR PET imaging of patients with newly diagnosed prostate cancer. Representative transverse CT, PET, and co-registered PET/CT images from the first-ever uPAR PET study in humans. *Upper panel* shows a primary tumor lesion (*blue arrow*) with high uptake of ^64^Cu-DOTA-AE105. uPAR immunohistochemistry on surgically removed prostate cancer tissue confirmed general pattern of uPAR expression. *Bottom* images show a uPAR-positive regional lymph node metastasis (*blue arrow*) with high ^64^Cu-DOTA-AE105 uptake. The subsequent staging operation and histopathological assessment confirmed prostate adenocarcinoma in three out of six removed lymph nodes. Reproduced from [44] with permission

As the first step towards clinical translation of uPAR PET imaging, we have conducted and reported the first-in-humans trial of ^64^Cu-DOTA-AE105 (ClinicalTrials.gov: NCT02139371). By definition, the primary endpoints of a phase I clinical study are safety, biodistribution, and dosimetry assessment based on three successive PET scans performed at 1, 3, and 24 h post injection. We included total of ten patients with urinary bladder (three patients), breast (three patients), and prostate cancer (four patients). Importantly, no adverse events or clinically detectable pharmacologic effects were found. Radiation dosimetry analysis estimated an effective dose of 0.0276 mSv/MBq, closely resembling the predicted effective dose from our previous mouse study [43], and equaling 5.5 mSv for a 200 MBq dose, which is lower/comparable radiation dose to the dose received from a standard FDG-PET [44]. Secondary objectives were to investigate the uptake in primary tumor lesions and potential metastases. Four patients with newly diagnosed and biopsy-proven PC (mean age 68, Gleason score 7–9) were uPAR PET scanned prior to surgical pelvic lymphadenonectomy for staging and prostatectomy if indicated. In all four patients, a high and specific uptake in the primary intraprostatic lesion was found (Fig. 3). Histopathological examination of three available surgical specimens confirmed a general pattern of uPAR expression in the primary tumor, supporting target-specific uptake of ^64^Cu-DOTA-AE105. One patient had several visible uPAR PET positive lymph nodes in the pelvic region, which was confirmed during the staging operation and the following histopathological assessment confirmed prostate adenocarcinoma in three out of six removed lymph nodes (Fig. 3). Two patients had no signs of metastases on neither uPAR PET nor perioperative staging, while the last patient was found to have a metastasis in 1 out of 17 regional lymph nodes that were not visualized on uPAR PET or CT. The results of this phase I study was encouraging with uPAR PET being able to identify both primary tumors and lymph node metastases in PC, although the limited number of patients precludes an evaluation of uPAR PET in the initial staging of PC. We have recently conducted another new phase I study, where safety, pharmacokinetics and dosimetry of a ^68^Ga-labeled version of AE105 (^68^Ga-NOTA-AE105) are being investigated in cancer patients (ClinicalTrials.gov: NCT02437539), and data are currently under evaluation.

## Future directions

PET imaging of uPAR expression seems to be highly promising and several important clinical questions in both primary and metastatic PC can potentially be addressed using uPAR PET.

In the diagnostic work up of patients suspected of PC, various imaging techniques have been suggested to enhance detection and localization of intraprostatic tumors [1]. The current guideline with transrectal/perineal core needle biopsies has a false negative rate of 20–25 % [45], and it is suggested that the use of specific molecular imaging might be helpful in image-guided biopsies, especially in patients with the previous negative findings [46]. However, since overtreatment is a big issue in localized PC, a huge clinical potential lies in the possibility of distinguishing indolent tumors from the invasive phenotype [44]. As noted above, uPAR expression correlates with PC aggressiveness. As such, it could be expected that with a quantitative imaging modality, such as PET, the degree of radiotracer uptake might correlate with pathological and clinical parameters, e.g., Gleason score and prognosis. Clinically, significant disease that would benefit from aggressive therapy with prostatectomy or radiotherapy instead of watchful-waiting could potentially be non-invasively identified by uPAR PET imaging.

Another important clinical implication of uPAR PET is pre-operative staging. The ability of uPAR PET to pre-operatively identify pelvic lymph node involvement in high-risk primary PC will have to be investigated in well-designed prospective studies. In addition, uPAR PET can be applied in the context of biochemical recurrence following failed local therapy (usually detected as a rise in serum PSA level). In these patients, a sensitive and reliable imaging assessment for the localization of the site of recurrent disease would potentially provide more appropriate guidance of treatment. Especially, in this indication, it will be relevant to perform a head-to-head comparison with the PSMA-targeting ligands that, in the recent years, have found widespread use in biochemical recurrence due to higher sensitivity than any other modality for relapse localization.

In addition, targeting of uPAR with a monoclonal antibody blocking the biologic functions of uPAR was, recently, shown to have a potent and encouraging therapeutic effect in murine prostate cancer models, including bone metastases formation [47]. A non-invasive method for specific assessment of tumor uPAR expression status would be valuable. Such a tool would be clinically relevant for the guidance of patient management and as companion diagnostics for emerging uPAR-targeting therapies.

**Fig. 4:**
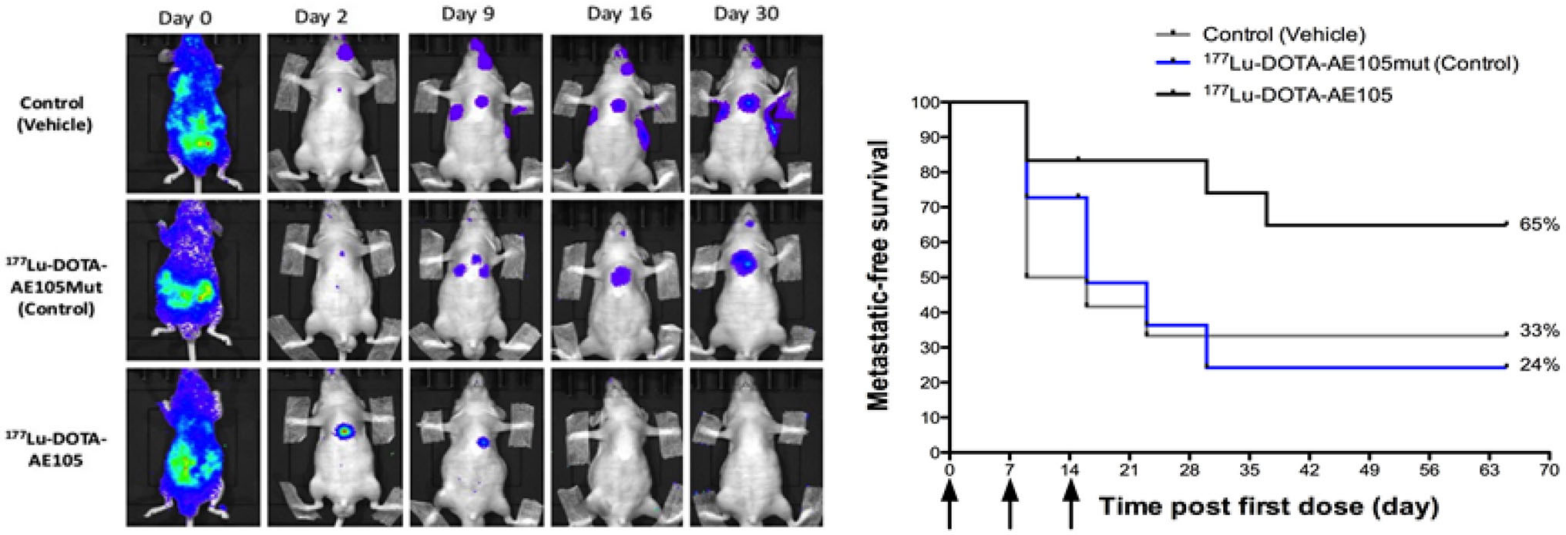
uPAR-targeted radionuclide therapy with ^177^Lu-DOTA-AE105 inhibits dissemination of metastatic prostate cancer. In a mouse model of disseminated prostate cancer (the same study as Fig. 2), the formation of small metastatic lesions was followed with bioluminecence imaging (BLI). The study investigated three groups (1, vehicle-treated controls; 2, control group treated with ^177^Lu-labeled non-binding control peptide; 3, treatment group receiving ^177^Lu-DOTA-A50150). Representative bioluminescence imaging for each of three treatment group during the 30 day-study depicts a clear tendency for an increased metastatic burden in both control groups (vehicle and ^177^Lu-DOTA-AE105mut) compared with the uPAR-targeted treatment group (^177^Lu-DOTA-AE105). A Kaplan–Meier plot shows the metastatic-free survival in each of the three treatment groups. Adapted with permission from [48]. Copyright 2014 American Chemical Society

An innovative and interesting perspective is to combine non-invasive PET imaging and targeted radionuclide therapy in the management of metastatic PC. In this setting, the same targeting ligand is radiolabeled with either a positron-emitting nuclide for PET imaging or an alpha/beta-emitter nuclide for therapeutic intervention. Such a dual functionality aligns excellently with the concept of personalized medicine [48]. Targeted radiotherapy has shown promising results in several cancers, with somatostatin receptor-based targeting of neuroendocrine tumors being the most successful so far [49], but also recently applied in PC with ^223^Ra (Zofigo™), an alpha-emitter, for treatment of bone-related pain in castration-resistant PC with bone metastases [50]. In fact, we have conducted two preclinical proof-of-concept studies with DOTA-AE105 conjugated with the beta-emitter ^177^Lu for uPAR-targeted radionuclide therapy in colorectal cancer [51] and in metastatic PC [48]. In metastatic PC (Fig. 4), we found a significant reduction in metastatic lesions and longer overall metastatic-free survival in mice treated with ^177^Lu-DOTA-AE105 compared to controls, thus setting the stage for a uPAR-mediated theranostic approach [48].

## Conclusion

Due to the importance in cancer invasion and metastatic development, uPAR is an attractive molecular target for non-invasive PET imaging in PC with the possibility of becoming a clinically relevant diagnostic and prognostic imaging biomarker. Several versions of uPAR-targeting PET ligands based on the high affinity peptide ligand AE105 have been synthesized and tested preclinically in human xenograft mouse models and, recently, also in a first-ever clinical uPAR PET study in humans that included also patients with PC. The clinical results, so far, are limited, but encouraging and support large-scale clinical trials to determine the utility of uPAR PET in the management of patients with PC with the goal of improving outcome.
